# Hypertensive Urgency With Primary Hyperaldosteronism Due to Unilateral Idiopathic Adrenal Hyperplasia: A Case of Headache With Severe Hypokalemia in a Young Male

**DOI:** 10.7759/cureus.74461

**Published:** 2024-11-25

**Authors:** Syed Abid, Humaira Jananth, Sayeda M Chowdhury, Tanveer Ahmed

**Affiliations:** 1 General Internal Medicine, East and North Hertfordshire NHS Trust, Stevenage, GBR; 2 Medicine, East and North Hertfordshire NHS Trust, Stevenage, GBR; 3 Internal Medicine, Evercare Hospital, Dhaka, BGD; 4 Critical Care, Evercare Hospital, Dhaka, BGD

**Keywords:** adrenal hyperplasia, adrenal imaging, aldosterone excess, electrolyte imbalance, hypertension, hypokalemia, mineralocorticoid antagonist, potassium supplementation, targeted treatment, young hypertensive

## Abstract

Hypertension remains a significant global health issue, increasingly affecting younger populations due to lifestyle and dietary changes. This case report presents a 28-year-old male diagnosed with primary hyperaldosteronism, a rare but treatable cause of secondary hypertension, presenting as hypertensive urgency. The patient reported persistent headaches and weakness, with an initial blood pressure of 190/120 mmHg and severe hypokalemia. Through a systematic diagnostic approach, including biochemical and imaging assessments, unilateral adrenal hyperplasia was identified as the cause of primary hyperaldosteronism. Targeted treatment, including antihypertensive agents, potassium supplementation, and mineralocorticoid receptor antagonists, led to significant improvements in blood pressure and electrolyte levels. This case underscores the importance of considering secondary hypertension causes in young adults and the potential for positive outcomes with accurate, resource-conscious diagnostic and management strategies.

## Introduction

Hypertension is one of the leading causes of global health burden, with millions affected by its complications, ranging from cardiovascular disease to renal failure [1]. The incidence of hypertension is alarmingly increasing, even among younger adults, partly due to lifestyle changes and dietary habits [2]. Hypertensive urgency - a condition marked by severe elevation in blood pressure without acute end-organ damage - necessitates prompt intervention to prevent potential progression to a hypertensive emergency, where organ damage can become imminent [3]. In low-resource settings, the management of such cases is often challenged by limited diagnostic tools, making clinical vigilance and comprehensive case assessment critical for effective care [4].

This case report presents a 28-year-old male patient, a garment worker, who was admitted with complaints of a persistent headache and generalized weakness over a period of 15 days. Despite being a young adult, his blood pressure readings were strikingly elevated. Initial management focused on assessing the underlying causes of this elevated blood pressure, given that secondary hypertension often has identifiable and treatable etiologies, particularly in younger patients [5]. Among these, primary hyperaldosteronism-characterized by excessive production of aldosterone leading to sodium retention, hypokalemia, and hypertension, is a notable cause that frequently goes undiagnosed despite its significant clinical implications [6].

The diagnostic approach for this patient incorporated a series of biochemical evaluations, including serum electrolytes, aldosterone, and plasma renin measurements, as well as imaging studies to assess adrenal function. Elevated serum aldosterone levels and findings on adrenal imaging ultimately confirmed a diagnosis of primary hyperaldosteronism attributed to unilateral adrenal hyperplasia. This is a rare but notable condition in young hypertensive patients and requires specific antihypertensive and potassium-sparing treatment to manage both blood pressure and electrolyte imbalances [7].

This report aims to underscore the importance of recognizing secondary causes of hypertension, especially in younger populations presenting with atypical features such as severe hypokalemia. In settings with limited resources, systematic and judicious use of available diagnostic tools can facilitate early and effective management, potentially preventing long-term complications and improving quality of life [8]. By detailing the diagnostic pathway, treatment plan, and outcomes of this case, this report seeks to contribute valuable insights into the management of hypertensive urgency associated with primary hyperaldosteronism.

## Case presentation

Problem statement

Hypertension, a major global health concern, increasingly affects younger populations, often leading to severe complications if undiagnosed or inadequately managed [1]. While primary hypertension is common, secondary hypertension, which has identifiable and treatable causes, remains under-recognized [2]. This case study of a young adult presenting with hypertensive urgency and primary hyperaldosteronism highlights the challenges in diagnosing secondary causes of hypertension. These conditions require specialized diagnostic approaches and targeted therapies to achieve optimal management and prevent long-term health complications [3].

Methodology

This case report outlines the clinical assessment, diagnostic approach, and management plan for a 28-year-old male patient presenting with hypertensive urgency at a tertiary hospital in September 2023. The methodology included the following steps:

On admission, a detailed history was taken to document the patient's presenting complaints, past medical history, family history, and social factors that could impact his health. The physical examination covered vital signs, general appearance, and a systematic evaluation of cardiovascular, neurological, and other systems to assess for potential end-organ damage associated with severe hypertension.

Routine blood tests were conducted to establish baseline health status, including a complete blood count, serum electrolyte levels, renal function tests, lipid profile, and blood glucose levels. Special attention was given to serum potassium levels, which is a key indicator in cases of suspected primary hyperaldosteronism, often associated with hypokalemia.

Given the findings of elevated blood pressure and hypokalemia, further investigations were carried out to explore potential secondary causes of hypertension. These included serum aldosterone concentration and plasma renin activity tests, then calculating the aldosterone-renin ratio. Imaging studies, such as an adrenal gland CT scan, were conducted to evaluate the possibility of adrenal hyperplasia or other abnormalities.

Following the diagnosis of primary hyperaldosteronism, the patient received a targeted treatment regimen focused on managing blood pressure and correcting electrolyte imbalances. Medications included antihypertensive agents, potassium supplementation, and mineralocorticoid receptor antagonists to address the underlying hormonal imbalance. The patient's response to treatment was carefully monitored, with follow-up blood pressure readings and electrolyte levels guiding any necessary adjustments.

 The patient was advised to attend monthly follow-up appointments for three months, during which serum electrolyte and renal function tests were repeated to monitor for any recurrence of hypokalemia and to ensure effective blood pressure management. Additionally, lifestyle counseling and adherence support were provided to promote sustained improvements in health outcomes and prevent future hypertensive episodes.

Results

A 28-year-old male patient presented with a two-week history of persistent headache and generalized weakness. Physical examination upon admission indicated hypertensive urgency, with blood pressure readings of 190/120 mmHg. Despite the severe elevation in blood pressure, there were no apparent signs of acute end-organ damage. Laboratory tests revealed profound hypokalemia, with serum potassium levels at 1.63 mmol/L (normal range: 3.5-5.0 mmol/L), suggesting an electrolyte disturbance potentially linked to secondary hypertension. The fundus examination was performed to rule out papilloedema, and the findings revealed no evidence of papilloedema.

This finding raised suspicion for primary hyperaldosteronism, as hypokalemia is a characteristic feature of this condition. The test reports of the patient are shown in Tables 1-9.

**Table 1:** Hematology report

Test	Result	Reference values
ESR (Westergren method)	5 mm in 1st hr	Male: 0-10 mm; Female: 0-20 mm in 1st hr
Total WBC count	7.51 x 10^9/L	4-11 x 10^9/L
Total RBC count	5.65 x 10^12/L	Male: 4.5 - 5.5 x 10^12/L; Female: 4.3 - 5.3 x 10^12/L
Total platelet count	279 x 10^9/L	150-450 x 10^9/L
Neutrophils	63%	40-70%
Lymphocytes	21%	20-40%
Monocytes	4%	2-8%
Eosinophils	12%	0-6%
Basophils	0%	< 1-2%
Haemoglobin	15.1 g/dl	Male: 13-17 g/dl; Female: 12-16 g/dl
Mean corpuscular volume	78.1 fL	76.00 - 96.00 fL
Mean corpuscular hemoglobin	26.7 pg	27 - 32 pg
Mean corpuscular hemoglobin concentration	34.2 g/dl	32.00 - 36.00 g/dl
Red cell distribution width-coefficient of variation	12.60%	11.0 - 14.0%
Mean platelet volume	9.9 fL	7.2 - 11.1 fL
Platelet large cell ratio	23.80%	13 - 43%
Plateletcrit	0.28%	0.01 - 0.09%
Platelet distribution width	11 fL	9 - 17 fL

**Table 2:** Comprehensive lab report

Variable	Result	Unit	Reference range
Potassium (K)	1.63	mmol/L	3.5-5.0
HbA1c	5.27	-	Diabetics: >6.5%; Good Control: <7.0%; Moderate Control: 7.0-8.0%; Poor Control: >8.0%
Magnesium	2.18	mg/dl	1.3-2.7
Sodium (Na)	141	mmol/L	135-145

**Table 3:** Kidney function and lipid profile test results HDL - high-density lipoprotein; LDL - low-density lipoprotein

Service/attribute	Result	Unit	Reference value
Kidney function			
Serum creatinine	1.31	mg/dL	0.60-1.3 mg/dL
Lipid profile			
Serum total - cholesterol	153	mg/dL	<200 mg/dL
Serum triglyceride	156	mg/dL	<150 mg/dL
Serum HDL - cholesterol	41	mg/dL	40-60 mg/dL
Serum LDL - cholesterol (direct)	87	mg/dL	<100 mg/dL

**Table 4:** Thyroid function test report TSH - thyroid stimulating hormone

Thyroid function	Result	Unit	Reference value
TSH	1.85	µIU/mL	Premature: 28-36wks: 0.7-27.0 Birth 2-4 days: 1.0-39.1 2-20wks: 1.7-9.1 21w-20y: 0.7-6.4 Adult 21-54y: 0.4-4.2 55-87y: 0.4-4.5
Free T4	1.24	ng/dL	Adults: 0.89-1.72 ng/dL

**Table 5:** Random blood sugar (RBS) report

Service/attribute	Result	Unit	Reference value
Random blood sugar (RBS)	55	mmol/L	< 7.8 mmol/L

**Table 6:** Urine analysis report

Urine analysis	Result	Unit	Reference range
Urine sodium (Na+)	43	mmol/L	60-220
Urine potassium (K+)	10.7	mmol/L	Oct-40
Urine chloride (Cl-)	51	mmol/L	60 - 200

**Table 7:** Aldosterone and renin concentration test report

Hormonal test	Result	Unit	Reference value
Aldosterone (direct renin concentration ratio)	1590	pmol/L	69.8 - 1085.8 pmol/L
Direct renin concentration	0.75	µIU/mL	4.4 - 46.1 µIU/mL
Aldosterone/renin ratio	2120:1	pmol/L/µIU/mL	>91 pmol/L/µIU/mL: Primary Aldosteronism probable

**Table 8:** M-mode and 2D echocardiographic measurements LA - left atrium; ACS - acute coronary syndrome; RVIDd - right ventricular dimension at end-diastole; IVSd - interventricular septum thickness at end-diastole; pWd - posterior wall diameter at end-diastole; MVA - mitral valve area; AV - aortic valve; AO - aorta; LVIDs - left ventricular internal dimension at end-systole; FS - fractional shortening; EF - ejection fraction; IVSs - interventricular septum thickness at end-systole; PWs - posterior wall diameter at end-systole; MV(EPSS) - mitral valve (E-point septal separation); E/A - peak velocity of early diastolic mitral annular motion / peak velocity of late transmitral flow; LVIDd - left  ventricular internal dimension at end-diastole

Measurement	Value	Unit
LA	36	mm
ACS	16	mm
RVIDd	0	mm
IVSd	10	mm
PWd	7	mm
MVA	0	cm²
AV (ring)	0	mm
AO	27	mm
LVIDs	27	mm
FS	32.5	%
EF	65	%
IVSs	0	mm
PWs	9	mm
MV (EPSS)	0	mm
E/A	Normal	
LVIDd	48	mm

**Table 9:** Abdominal ultrasound findings

Organs	Findings
Gallbladder	Normal size. Wall not thickened. No gallstones or sludge.
Liver	Normal size (14.85 cm). Normal echogenicity (texture). No lesions.
Pancreas	Normal in size and texture. Main pancreatic duct not dilated.
Spleen	Normal size (12.39 cm) and texture.
Right kidney	Normal size, shape, and position. No stones or masses.
Left kidney	Normal size, shape, and position. No stones or masses.
Urinary bladder	Normal wall thickness. No lesions inside.
Prostate	Normal size (3.34 x 4.61 x 3.11 cm), shape, and texture. Volume about 17.4 ml.

The patient underwent a series of investigations, including an electrocardiogram (ECG), an abdominal ultrasound, and an echocardiogram. The ECG revealed a regular heart rhythm with no abnormalities detected, indicating normal electrical activity and blood flow. The abdominal ultrasound showed all organs within normal limits, with no pathological findings. Similarly, the echocardiogram demonstrated normal cardiac function with no abnormalities in the measurements. Based on these results, no specific regions of interest (ROIs) were identified in any of the reports, suggesting no further investigation is warranted at this time.

Routine laboratory evaluations, including complete blood count, renal function tests, fasting lipid profile, and blood glucose, largely fell within normal limits, indicating no underlying renal or metabolic conditions contributing to hypertension. However, targeted hormonal studies showed a significantly elevated serum aldosterone concentration of 1590 pmol/L (reference range: 69.8-1085.8 pmol/L) and a correspondingly low plasma renin activity of 0.75 µIU/mL. The resultant aldosterone-renin ratio was substantially elevated, a hallmark diagnostic criterion for primary hyperaldosteronism. This hormonal imbalance supported the hypothesis of an aldosterone-mediated mechanism driving both the patient's hypertension and hypokalemia.

The urine analysis report indicates that the sample is within normal ranges, with no concerning findings across physical, chemical, or microscopic examinations. Physically, the urine is a healthy straw color, clear in appearance, and contains no sediment, which is typical of a healthy individual. Chemically, the specific gravity of 1.010 is within the normal range, indicating normal urine concentration, while the pH of 6.0 is mildly acidic, which is also normal. No albumin, sugar, ketones, nitrites, urobilinogen, or bilirubin were detected, all of which would normally be absent in healthy urine and indicate good kidney function without infection or metabolic abnormalities. The potassium (K) level measured in the subject's blood was found to be 1.63 mmol/L, which is below the ideal reference range of 3.5-5.0 mmol/L. This lower value has been highlighted in bold to emphasize its clinical significance. Potassium is a crucial electrolyte involved in various physiological functions, including maintaining proper cell function, nerve transmission, and muscle contraction. A potassium level lower than the normal range can be indicative of hypokalemia, which may result from factors such as dehydration, excessive use of diuretics, or certain medical conditions (Table 2).

Microscopic examination further supports these findings: there are minimal pus cells (0-2 per high power field) and epithelial cells (0-3 per high power field), which are within normal limits, with no red blood cells present. Additionally, no crystals (like calcium oxalate or uric acid) or casts, which could signal kidney stones or kidney disease, were found. The absence of yeast, Trichomonas vaginalis, and other microorganisms indicates no infection. Overall, this report suggests a healthy urinary system, free from infection, inflammation, and other abnormalities.

To further investigate the adrenal etiology, a computed tomography (CT) scan of the adrenal glands was performed. Imaging results revealed mild unilateral enlargement of the left adrenal gland, consistent with adrenal hyperplasia, a structural anomaly often associated with aldosterone overproduction. The combination of biochemical evidence of hyperaldosteronism with unilateral adrenal hyperplasia on imaging provided a robust basis for diagnosing primary hyperaldosteronism, a known cause of secondary hypertension. This diagnosis is essential because it enables the implementation of targeted therapy, which can result in significant blood pressure control and correction of hypokalemia.

The patient's treatment plan included a combination of antihypertensive and potassium-sparing therapies. A regimen of Amlodipine (a calcium channel blocker) and Olmesartan (an angiotensin II receptor blocker) was initiated to manage blood pressure. Additionally, spironolactone, a mineralocorticoid receptor antagonist, was prescribed to counteract the effects of aldosterone excess and help maintain potassium balance. Potassium chloride supplements were also administered to correct the hypokalemia. Given the initial severity of hypertension, a beta-blocker (labetalol) was included to provide additional blood pressure control and mitigate any adrenergic symptoms.

Within a few days of initiating therapy, the patient's blood pressure decreased significantly, stabilizing to levels below 140/90 mmHg. Concurrently, his serum potassium levels gradually improved, reaching near-normal levels within the first week. This positive response highlighted the effectiveness of a treatment approach tailored to address primary hyperaldosteronism, showcasing the potential for rapid and sustained improvement in blood pressure and electrolyte status with appropriate medical intervention.

The patient was scheduled for monthly follow-up visits over a three-month period to monitor blood pressure and serum electrolyte levels. At each follow-up, blood pressure remained within the target range, and no signs of recurrent hypokalemia were observed. Serum potassium levels were consistently within normal limits, reflecting sustained control of the hormonal imbalance. Throughout the follow-up period, the patient demonstrated good adherence to the medication regimen, and his general well-being significantly improved, with no further episodes of headache or generalized weakness reported.

These follow-up results underscore the importance of continued monitoring in cases of secondary hypertension due to primary hyperaldosteronism. Regular assessment ensured that any deviations in blood pressure or potassium levels could be promptly addressed, preventing potential complications. This case demonstrates the successful management of hypertensive urgency with primary hyperaldosteronism through a systematic approach that incorporates early diagnosis, targeted therapy, and diligent follow-up care, which is crucial for achieving long-term stability and improving patient outcomes.

## Discussion

This case report underscores the complexities and challenges in managing hypertensive urgency in young adults, particularly within resource-limited settings. The diagnostic journey highlighted the importance of considering secondary hypertension in younger patients presenting with atypical signs, such as hypokalemia. Primary hyperaldosteronism, though rare, emerged as a critical underlying factor, emphasizing the role of comprehensive biochemical and imaging assessments in confirming the diagnosis. The patient's significant clinical improvement following targeted therapy with mineralocorticoid receptor antagonists, potassium supplements, and antihypertensive agents demonstrates the efficacy of a tailored therapeutic approach. This case also illustrates the need for increased awareness and diagnostic vigilance for secondary hypertension causes, especially in healthcare environments with limited resources. Through a systematic and diligent approach, effective management and monitoring can lead to rapid stabilization and improved outcomes in similar cases.

## Conclusions

This case study emphasizes the critical role of recognizing secondary hypertension in young adults with hypertensive urgency, particularly in settings with limited access to diagnostic resources. In this instance, primary hyperaldosteronism, though rare, was identified through a methodical approach that included clinical evaluation and laboratory tests. The patient's condition was successfully managed with targeted medications and ongoing monitoring, leading to a positive recovery. The case serves as a reminder for healthcare providers to implement thorough diagnostic protocols when treating young patients with high blood pressure, as early detection and treatment can significantly improve long-term outcomes and quality of life.
